# Increased Low-Frequency Resting-State Brain Activity by High-Frequency Repetitive TMS on the Left Dorsolateral Prefrontal Cortex

**DOI:** 10.3389/fpsyg.2017.02266

**Published:** 2017-12-22

**Authors:** Shao-Wei Xue, Yonghu Guo, Wei Peng, Jian Zhang, Da Chang, Yu-Feng Zang, Ze Wang

**Affiliations:** ^1^Institutes of Psychological Sciences, Hangzhou Normal University, Hangzhou, China; ^2^Center for Cognition and Brain Disorders, Hangzhou Normal University, Hangzhou, China; ^3^Zhejiang Key Laboratory for Research in Assessment of Cognitive Impairments, Hangzhou, China; ^4^Department of Radiology, Lewis Katz School of Medicine, Temple University, Philadelphia, PA, United States

**Keywords:** transcranial magnetic stimulation (TMS), functional magnetic resonance imaging, anterior cingulate cortex, dorsolateral prefrontal cortex, default mode network

## Abstract

Beneficial effects of repetitive transcranial magnetic stimulation (rTMS) on left dorsolateral prefrontal cortex (DLPFC) have been consistently shown for treating various neuropsychiatrical or neuropsychological disorders, but relatively little is known about its neural mechanisms. Here we conducted a randomized, double-blind, SHAM-controlled study to assess the effects of high-frequency left DLPFC rTMS on resting-state activity. Thirty-eight young healthy subjects received two sessions of either real rTMS (*N =* 18, 90% motor-threshold; left DLPFC at 20 Hz) or SHAM TMS (*N =* 20) and functional magnetic resonance imaging scan during rest in 2 days separated by 48 h. Resting-state bran activity was measured with the fractional amplitude of low-frequency fluctuation (fALFF) and functional connectivity (FC). Increased fALFF was found in rostral anterior cingulate cortex (rACC) after 20 Hz rTMS, while no changes were observed after SHAM stimulation. Using the suprathreshold rACC cluster as the seed, increased FC was found in left temporal cortex (stimulation vs. group interaction). These data suggest that high-frequency rTMS on left DLPFC enhances low-frequency resting-state brain activity in the target site and remote sites as reflected by fALFF and FC.

## Introduction

Transcranial magnetic stimulation (TMS) (Barker et al., 1985) is a non-invasive neuromodulational tool that has been widely used in cognitive neuroscience research (Bolognini and Ro, 2010) as well as neuropsychological or psychiatric disease studies (Fregni and Pascual-Leone, 2007; Rossini and Rossi, 2007). TMS relies on a changing magnetic field generated by quickly and frequently charging and discharging a capacitor. The magnetic field can penetrate the scalp and skull with negligible loss and subsequently induce a changing electrical field and the associated electrical currents in the superficial brain cortex (Hallett, 2000; Hoogendam et al., 2010). Through the interactions between the induced currents and neuronal electric activity, TMS can temporarily disrupt the ongoing cortical activity, leading to macroscopic deactivations or excitations of the affected brain regions (Allen et al., 2007). While such transit alterations usually only sustain for a short time after turning off TMS, the repetitive application of TMS (rTMS) has been shown to have long-lasting effects (Hallett, 2007), which has provoked an increasing application of TMS in treating various neuropsychiatrical or neuropsychological disorders (Trojak et al., 2015; Lan et al., 2016).

In contrast to the increasing popularity of TMS in both the neuroscientific research and clinical applications, relatively little is known about their brain mechanisms. “Virtual lesion” is a traditional concept often used to design and explain TMS effects. During such experiments, single pulse TMS or paired pulse TMS are used to temporally change the ongoing brain activity to observe the corresponding behavioral consequence (Siebner et al., 2009), similar to the traditional brain-lesion-based brain function mapping paradigm. Such “lesion”-like effects may be attributed to the pre-synaptic excitation of specific subsets of neurons or a post-synaptic inhibition in cortex stimulated by the changing electrical field. The preferential axon excitation induces artificial neural synchronization (Bestmann, 2008; Miniussi et al., 2010), while the prolonged post-synaptic potential leads to a blockage of transmission of action potential and the associated message (Reithler et al., 2011). Either factor or a combination of both (excitability and inhibition) may eventually lead to the temporary virtual lesion effects. These transit consequences may be prolonged after a repetitive application of magnetic stimulations in rTMS. While the mechanisms underlying the long-term effects of rTMS still remain elusive, researchers have found that rTMS shares several common features with the long-term potentiation/depression (LTP/LTD) (Bliss and Lomo, 1973) of the excitatory synaptic transmission (Hoogendam et al., 2010; Pell et al., 2011). Both rTMS and LTP/LTD are sensitive to the temporal pattern of the stimulation protocol, dependent on the induced excitability changes on the preceding activation history (Abraham and Bear, 1996), and dependent on the initiation and maintenance of synaptic plasticity (such as gene and protein expression, NMDA receptor functioning). However, rTMS and LTP/LTD still differ in many other ways (Hoogendam et al., 2010; Pell et al., 2011), making it questionable to claim the sharing of the same underlying neuronal mechanisms. Away from the virtual-lesion assumption, TMS has been treated as a means to insert information into the ongoing brain activity and that interaction may depend on the specific TMS parameters and pre-conditions of brain states as shown in various studies (Silvanto et al., 2008; Pasley et al., 2009; Reithler et al., 2011; Romei et al., 2016). Meanwhile, TMS effects have been considered as results of adding stochastic noise to neuronal processing (Schwarzkopf et al., 2011).

To understanding the neuronal effects of TMS, one needs to probe the brain signal with and without TMS. Over the past decade, many tools have been used to that endeavor, among them neuroimaging, especially functional magnetic resonance imaging (fMRI), provides a versatile tool to directly map the effects in the entire brain (Liston et al., 2014; Nettekoven et al., 2014; Salomons et al., 2014). Their combined use, sometimes called as the “perturb-and-measure” approach (Ruff et al., 2009), can be used to examine functional interactions between different brain areas and functional cortical plasticity in either an online mode or an offline mode (Grefkes et al., 2010). The online rTMS–fMRI can evaluate the acute effect of magnetic stimulation, and the offline mode is generally used to assess the long-term effects of rTMS and is more widely adopted in neuroscience research. The purpose of this study was to assess the offline high-frequency rTMS effects on resting-state brain activity in normal healthy adult brain. We focused on resting-state brain activity because it is a major type of brain activity that accounts for most of brain energy consumption (Zhang and Raichle, 2010). Because high-frequency rTMS is often cited for its excitatory effects (Wobrock et al., 2015), we hypothesized that high-frequency rTMS would increase resting-state brain activity especially for the most commonly cited low-frequency components. Left dorsolateral prefrontal cortex (DLPFC) was chosen as the TMS target site because it is the most widely used one in the literature. DLPFC is a key element of many high-order brain functions including inhibition control, attention, working memory, and decision-making (O’Reilly, 2010). Stimulating DLPFC using the beneficial high-frequency rTMS may then help improve these complex functions and subsequently improve the associated brain disease condition such as depression (Pascual-Leone et al., 1996; George and Post, 2011; Mylius et al., 2013; Lefaucheur et al., 2014). Moreover, DLPFC is both structurally and functionally connected to many cortical and sub-cortical regions, stimulating DLPFC can also provide a means to assess the remote effects of rTMS through either connectivity or network-wise interactions (Fox et al., 2012).

## Materials and Methods

### Participants

This study was carried out in accordance with the recommendations of local IRB in Hangzhou Normal University. All subjects signed written consent forms before participating in any experiment. The consent form and the study were in accordance with the Declaration of Helsinki. Thirty-eight young healthy subjects (age = 22.87 ± 2.83 years; 16 males) were randomly assigned to one of the two groups: SHAM or rTMS. Two sessions of MRI scans were performed on two separate days with 48 h apart using the same imaging protocol. The MRI scan after stimulation (rTMS or SHAM) was performed right after rTMS (post-rTMS) or SHAM stimulation (post-SHAM) (within 15 min due to the pre-scan preparations) to ensure that the stimulation effects are measured (Siebner et al., 2009). The 48 h interval between the two sessions was chosen to avoid any residual rTMS or SHAM effects from the preceding session if the stimulations were applied therein. All subjects were right-handed and reported no history of neurological or psychiatric disorders More demographic of the participants are summarized in **Table 1**.

**Table 1:** Demographic information of the subjects.

	rTMS	SHAM	*p*-value
Number of subjects	18	20	
Age (years)	22.44 ± 2.20	23.25 ± 3.31	0.29
Age range (years)	18–28	18–30	
Gender (M/F)	9/9	7/13	

*The number in age consists of mean and standard deviation. rTMS, repetitive transcranial magnetic stimulation; SHAM, SHAM rTMS.*

### rTMS

Repetitive transcranial magnetic stimulation was performed with the Magstim Rapid stimulator (Magstim Ltd., Whitland, United Kingdom) with a figure-of-eight coil. Neuronavigation was performed with the Brainsight Frameless Stereotaxic System (Magstim Ltd., Whitland, United Kingdom) which can dynamically visualize the TMS coil position on top of the individual subjects’ structural MRI. The following steps were used to find the target left DLPFC spot for each individual brain: (1) skull stripping for the high-resolution structural MRI and registering them into the Montreal Neurological Institutes (MNI) standard brain space; (2) 3D brain reconstruction and locating the target spot on the surface of the reconstructed brain cortex using the coordinate in MNI space [here the left DLPFC coordinate was set to be (-40, 26, 37)] (Guse et al., 2010); and (3) locating the projected spot on the subject’s brain scalp using the 3D neuronavigator.

Repetitive transcranial magnetic stimulation was applied following the safety guidance provided by the International Workshop on the Safety of Repetitive Transcranial Magnetic Stimulator (Wassermann, 1998). rTMS was administered in 12 successive pulse blocks interleaved with 28 s quite time. Each block consisted of 50 pulses with 20 pulses per second (20 Hz) for 2.5 s. The magnitude of pulse was set to be 90% of the resting motor threshold. The same pulse train was used for SHAM stimulation except that the coil was reoriented to be orthogonal to the direction of rTMS, i.e., orthogonal to the local surface of skull. No subject reported any strange feelings or aversive effects including pain except the pulse noise during rTMS or SHAM stimulation.

### MRI Data Acquisition

Magnetic resonance imaging scans were performed in a 3T Discovery MR 750 Scanner (General Electric, Waukesha, WI, United States) at the Center for Cognition and Brain Disorders at Hangzhou Normal University, China. During the scan, a comfortable and tight cushion was placed to immobilize the head and reduce motion. The participants were instructed to relax and remain still with their eyes open, not to fall asleep, and not to think about anything in particular. A gray screen with a black crosshair in the middle was used for eye fixation. All subjects were monitored through the video camera in the scanner room and nobody was found to fall asleep during the scan, which was also confirmed by interview after the scan.

Both structural MRI and resting-state functional MRI (rsfMRI) were acquired. High-resolution T1-weighted structural MRI was acquired with a 3D spoiled gradient (3D SPGR) echo sequence with repetition time/echo time (TR/TE) = 8.1/3.39 ms, flip angle = 7°, field of view = 256 × 256 mm, matrix = 256 × 256, 1.0 mm^3^ isotropic voxels, and 176 slices without interslice gap. Resting fMRI was acquired with a T2^∗^-weighted gradient-echo EPI pulse sequence with the following parameters: TR/TE = 2000/30 ms, flip angle = 90°, field of view = 220 × 220 mm, matrix = 64 × 64, 3.4 mm^3^ isotropic spatial resolution, and 37 interleaved slices. One hundred and eighty images were acquired in 6 min.

### MR Image Preprocessing

Statistical Parametric Mapping (SPM8, Wellcome Department, London, United Kingdom^1^) and an SPM8-based package, Data Processing Assistant for rsfMRI (Yan and Zang, 2010) (DPARSF^2^) were used for MR image processing. For each subject, the first 10 rsfMRI volumes were discarded to allow the mean magnetization to reach steady state and the participants to get familiar to the MR scan environment. The remained rsfMRI data were then corrected for the acquisition time difference between the 2D image slices, and were realigned to the first volume to correct head-motions. The maximum translational motion of all subjects was less than 2 mm and the maximum rotation was less than 2°. rsfMRI images registered with the T1-weighted structural MRI and subsequently registered into the MNI standard space using the transform defined based on the registration process of the T1-weighted MRI (to the MNI space). They were then smoothed with an isotropic 3D Gaussian kernel with a full width at half maximum of 8 mm^3^.

### fALFF Analysis

Fractional amplitude of low-frequency fluctuation (fALFF) was calculated from the preprocessed rsfMRI images at each intracranial voxel as the ratio of the power spectrum of the low-frequency sub-band (0.01–0.08 Hz) to the power of the entire acquired frequency band. fALFF map was then standardized by subtracting the mean and dividing by the standard deviation to reduce the global effects of variability across participants for statistical analysis. A two sample *t*-test was performed to examine the possible fALFF difference of the pre-stimulation condition between the rTMS and SHAM cohort. Paired *t*-tests were then used to statistically infer the within-subject pre- and post-rTMS or SHAM fALFF difference. A two-sample *t*-test was also performed to infer the between group (rTMS vs. SHAM) pre- and post-stimulation fALFF difference. Statistical significance of the analysis results was defined by *p* < 0.005 at the voxel level and cluster size >46 at the cluster level [corrected for multiple comparison using the Monte-Carlo simulation-based approach as implemented in AlphaSim (Ledberg et al., 1998)]. Image smoothness was FWHM of (9.4, 9.4, and 9.2 mm) estimated from the processed images.

### Functional Connectivity Analysis

As fALFF only infers regional brain activity, to examine the inter-regional activity changes in response to rTMS, we performed functional connectivity (FC) analysis using the same preprocessed data particularly because FC may represent a mechanism underlying the remote effects of rTMS (Fox and Raichle, 2007; Fox et al., 2012; Saiote et al., 2013). We focused on FC of any part of the brain to regions with significant rTMS fALFF effects identified in above analysis by using those regions as the seeds. FC was calculated as the correlation coefficient between the mean time-course of the seed region and the assessed voxel. Fisher *Z* transform was used to convert the correlation coefficient map into a *z* map, and the within subject stimulation effects and across-group rTMS vs. SHAM FC change difference were then assessed using paired *t*-test and two-sample *t*-test similar to those used for fALFF analysis. A two-sample *t*-test was performed to infer the between group (rTMS vs. SHAM) pre- vs. post-stimulation FC difference.

## Results

**FIGURE 1:**
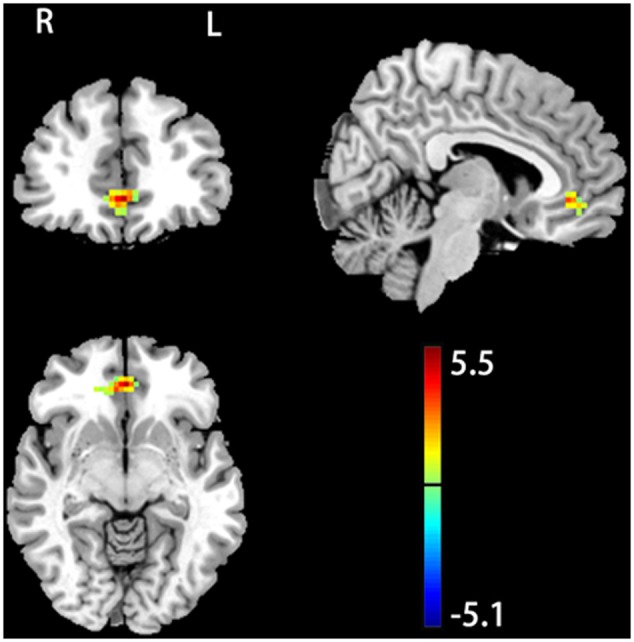
20 Hz rTMS induced change of fractional amplitude of low-frequency fluctuation (fALFF). Color bar represents *t*-score values. The warm and cold colors represent higher and lower fALFF after rTMS, respectively. Significance level was defined at *p* < 0.005, cluster size > 46 voxels, AlphaSim corrected. The left side of the image corresponds to the right side of the brain.

No significant baseline (pre-stimulation condition) fALFF difference was observed between the two groups. rTMS induced significant fALFF changes but SHAM didn’t. **Figure 1** shows the post-rTMS vs. pre-rTMS fALFF comparison results. Significant fALFF increase after 20 Hz left DLPFC rTMS was found in rostral anterior cingulate cortex (rACC) and ventro-medial prefrontal cortex (vmPFC) (peak MNI coordinates: *x* = 0, *y* = 42, *z* = -6). The between group post–pre stimulation delta fALFF comparison didn’t show any significant difference.

**FIGURE 2:**
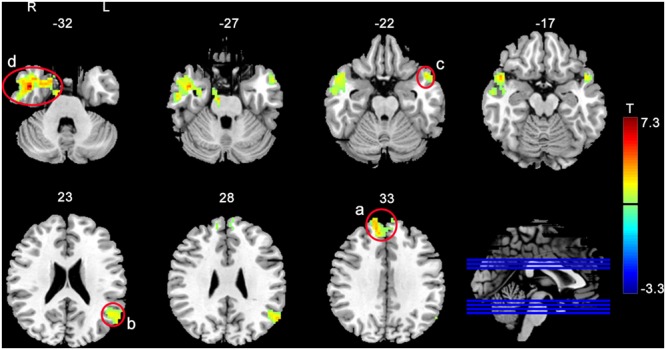
Increased rACC–FC after 20 Hz left DLPFC rTMS. The significance level was defined at *p* < 0.005, cluster size > 46 voxels, AlphaSim corrected. Color bar represents *t*-values. The warm and cold colors represent higher and lower FC difference, respectively.

Functional connectivity analysis was based on the rACC/vmPFC seed as defined by the aforementioned suprathreshold post- vs. pre-rTMS fALFF difference analysis. **Figure 2** and **Table 2** show the seed-based FC analysis results. No significant baseline rACC/vmPFC-FC (simplified as rACC–FC hereafter) difference was observed between the rTMS and SHAM group. Increased rACC–FC after 20 Hz rTMS (**Figure 2** and **Table 2**) was found in right medial superior frontal gyrus, left angular gyrus, left superior temporal pole, and right inferior temporal gyrus. SHAM didn’t show any significant rACC–FC changes.

**Table 2:** Regions showing altered rACC–FC after rTMS.

Zone	Brain region	BA	MNI (*X Y Z*)			Peak *t*-value	Cluster size (mm^3^)
a	Sup. med. frontal gyrus L	9	–3	42	42	4.59	6129
a	Sup. med. frontal gyrus R	32	12	51	30	5.50	4995
a	Sup. frontal gyrus R	9	15	57	36	4.86	675
b	Angular L	N/A	–57	–63	27	4.25	729
b	Mid. temporal gyrus L	39	–48	–57	24	3.88	972
c	Sup. temporal pole L	38	–51	12	–21	4.05	459
c	Mid. temporal gyrus L	N/A	–54	9	–24	3.75	378
c	Mid. temporal pole L	38	–51	12	–24	3.83	324
c	Inf. temporal gyrus L	20	–39	–6	–33	4.1	405
d	Inf. temporal gyrus R	20	42	0	–36	5.98	4347
d	Parahippocampal R	36	30	3	–33	5.53	891
d	Fusiform R	36	30	3	–36	4.41	594
d	Sup. temporal pole R	38	51	9	–15	4.58	648
d	Mid. temporal gyrus R	20	45	0	–27	4.83	2565
d	Mid. temporal pole R	N/A	21	6	–36	5.61	2511

*BA, Brodmann’s area; R, right side; L, left side; inf., inferior; sup., superior; Mid., middle; MNI, Montreal Neurological Institute coordinates. Significance level was defined at *p* < 0.005, cluster size > 46 voxels, AlphaSim corrected.*

**FIGURE 3:**
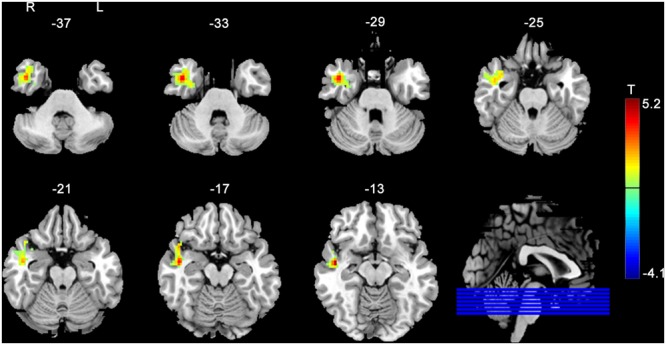
rACC–FC changes due to rTMS as compared to SHAM stimulation. The rACC–FC difference was measured between the post-rTMS minus pre-rTMS and the post-SHAM minus pre-SHAM. The warm and cold colors indicate positive and negative *t*-score values, respectively. Significance level was defined with a voxel-wise *p* < 0.005, cluster size > 46 voxels with AlphaSim corrected.

**Figure 3** shows the between group post- vs. pre-stimulation FC difference comparison results. As compared to SHAM, 20 Hz rTMS yielded greater FC increase in temporal cortex and hippocampus.

## Discussion

We assessed effects of left DLPFC high-frequency rTMS on resting-state brain activity regarding the low-frequency fluctuations and inter-regional FC. Our results showed significantly increased fALFF in rACC/vmPFC after rTMS but not after SHAM stimulation, which proves truth of our hypothesis about the beneficial high-frequency rTMS on resting-state brain activity.

Rostral anterior cingulate cortex/ventro-medial prefrontal cortex is involved in many important brain functions, including self-regulation, self-referential processing, decision-making, and emotion regulation (Miller, 2000; Posner et al., 2007; Rangel et al., 2008; Fuster, 2009; Spreng et al., 2009; Etkin et al., 2011), and has been involved in a variety of psychiatric diseases or brain disorders including schizophrenia, drug addiction, and depression, etc. Increased resting fALFF after 20 Hz rTMS in rACC/vmPFC suggests that high-frequency rTMS on left DLPFC may help enhance the aforementioned brain functions. In brain disorders, increased rACC/vmPFC activity may lead to a better treatment response. As shown in a depression meta-analysis study, higher rACC activity during task-performance or resting-state before treatment predicted better treatment response in depression (Pizzagalli, 2011). rACC/vmPFC is also a central part of the default mode network (DMN) (Raichle et al., 2001), which persistently interplays with the task positive network (TPN) consisting of DLPFC, dorsal ACC, medial temporal area, and other brain regions (Corbetta and Shulman, 2002). In summary, our findings of increased fALFF only in rACC/vmPFC may underlie the effectiveness of left DLPFC high-frequency rTMS on various psychiatric disorders.

Our subsequent FC analysis revealed enhanced rACC/vmPFC FC to frontal and temporal regions, suggesting rACC as a pivotal node in DMN. While SHAM stimulations didn’t yield significant fALFF changes or inter-regional FC changes, the rTMS-induced fALFF changes were not significantly different from the post-SHAM vs. pre-SHAM fALFF changes. This “no-show” may be due to the large individual level variations often seen in rTMS (Hamada et al., 2012; Hinder et al., 2014; López-Alonso et al., 2014). Larger sample size and more personalized stimulation parameter tuning may be required to identify the rTMS vs. SHAM difference.

Repetitive transcranial magnetic stimulation increased FC between rACC/vmPFC and a few brain regions within DMN, including medial prefrontal cortex, temporal cortex, and parietal cortex. Increased rACC/vmPFC-FC was still observed in temporal cortex and hippocampus after controlling the placebo effects (SHAM stimulation). These findings suggest rACC/vmPFC as a hub region for facilitating the left DLPFC rTMS effects likely by first projecting its effects (or injecting information following the information theory-based rTMS effect model (Silvanto et al., 2008; Pasley et al., 2009; Reithler et al., 2011; Schwarzkopf et al., 2011; Romei et al., 2016) into rACC/vmPFC through the frontal-cingulate pathway and then spreading into the other DMN areas through the within-DMN connectivity. Since we didn’t find any significant rTMS-induced change to FC between rACC/vmPFC and left DLPFC, the DLPFC rTMS effects on rACC/vmPFC might then occur via the structural connectivity between DLPFC and rACC or through a network-wise interaction between DMN and the TPN, with which rACC/vmPFC and DLPFC are associated, respectively.

Several limitations should be mentioned in this study. The sample size included was moderate and the SHAM stimulation and rTMS were applied to separate cohorts, both may contribute large variations to the observed effects and may explain some of the no-show effects as mentioned above. Future studies with larger sample sizes would be necessary to fully evaluate the potential confounding effect of rTMS. Additionally, many parameters can affect rTMS effects, including frequency, intensity, and pre-conditions (Pell et al., 2011; Schwarzkopf et al., 2011), but we only assessed the effects of a fixed frequency (20 Hz) and intensity in this study, so our data couldn’t provide inferences to the variations due to those parameters. The intensity of TMS was 90% of the motor threshold. We chose this threshold to reduce the possible pain caused by the coil vibration, which surely is empirical. Using different intensity might change the results but that needs future investigation.

## Conclusion

In summary, left DLPFC rTMS induced increased rACC fALFF and increased rACC–FC to other components of the DMN, suggesting a possible neuronal mechanism of the well-observed beneficial effects of DLPFC high-frequency rTMS.

## Ethics Statement

This study was carried out in accordance with the recommendations of the Human Research Ethics Committee of Hangzhou Normal University with written informed consent from all subjects. All subjects gave written informed consent in accordance with the Declaration of Helsinki. The protocol was approved by the Human Research Ethics Committee of Hangzhou Normal University

## Author Contributions

The contributions of each author meet the full criteria issued by the International Committee of Medical Journal Editors. ZW and Y-FZ conceived and designed the experiments. WP, JZ, and DC performed the experiments. YG and S-WX analyzed the data and summarized the results. S-WX and ZW organized the manuscript and wrote the first draft of the manuscript. S-WX and ZW finalized the report.

## Conflict of Interest Statement

The authors declare that the research was conducted in the absence of any commercial or financial relationships that could be construed as a potential conflict of interest.

## Footnotes

1 http://www.fil.ion.ucl.ac.uk/spm

2 www.restfmri.net
